# Fibroblast-mediated uncaging of cancer cells and dynamic evolution of the physical microenvironment

**DOI:** 10.1038/s41598-021-03134-w

**Published:** 2022-01-17

**Authors:** Chang Liu, Michael Mak

**Affiliations:** Biomedical Engineering Department, Yale University, New Haven, 06511 USA

**Keywords:** Cancer, Cancer microenvironment, Cancer models, Metastasis

## Abstract

Stromal cells are prominent in solid tumor microenvironments and contribute to tumor progression. In particular, fibroblasts are common cell types in the tumor stroma that play important roles in remodeling the extracellular matrix (ECM). Here, we perform co-culture experiments with tumor cells and fibroblasts embedded in 3D collagen I matrices. We investigate the impact of fibroblasts on the migratory behavior of neighboring tumor cells and on the evolution of the surrounding ECM. We find that fibroblasts increase tumor cell motility and facilitate the transition from confined to diffusive tumor cell motions, indicative of an uncaging effect. Furthermore, the ECM is globally and locally remodeled substantially with the presence of fibroblasts. Moreover, these fibroblast-mediated phenomena are in part dependent on matrix metalloproteinases.

## Introduction

The solid tumor microenvironment (TME) is highly complex, typically consisting of heterotypic cell populations within a 3D fibrillar extracellular matrix (ECM). The ECM can be dense, particularly in fibrotic tumors often observed in many solid cancer types (e.g. in breast, lung, liver, pancreas)^1^. The dense ECM, which consists of small pores, poses as a biophysical barrier against cell migration^2^. During tumor progression and metastasis, this physical barrier is overcome. Certain canonical mechanisms for overcoming the ECM barrier have been established, including secretion of matrix metalloproteinases (MMPs) by tumor cells to degrade ECM fibers^2,3^ and transition of tumor cells to an amoeboid migratory mode, which relies on cell squeezing through confined spaces via actomyosin-dependent contractile processes^4,5^. Additional mechanisms are emerging in recent studies. The ECM can be remodeled by cells via mechanical forces due to the viscoplasticity of the matrix material^6–9^. Stromal cells can also facilitate tumor progression and invasion in a variety of ways^10,11^.

Recent studies have shown that fibroblasts are capable of leading tumor cells out of the primary tumor to initiate dissemination^12^, and that this process is associated with adhesions between tumor cells and fibroblasts. However, fibroblasts are capable of performing many additional functions that may contribute to invasion, including remodeling the ECM and generating biophysical cues^13^. Contractile cells can generate locally aligned topographies and ECM stiffening^14,15^, which are known to induce increased tumor invasiveness^16–18^. Additionally, fibroblasts can degrade the ECM through MMPs, which can create more space for migration^19,20^. The full effects of fibroblasts on tumor cell behavior and on the spatiotemporal evolution of the TME are not completely elucidated.

Here, we investigate the migration profiles of tumor cells with and without the presence of neighboring fibroblasts inside a 3D ECM. We use MDA-MB-231 breast cancer cells and primary human lung fibroblasts (NHLFs). MDA-MB-231s are metastatic solid tumor cells, and NHLFs represent fibroblasts found in normal stromal tissue. For ECM, we use collagen I, which is one of the most prominent ECM proteins in connective tissues and in the solid tumor microenvironment^21–23^. As the fibroblast is the most abundant stromal cell type in many solid tumors, and the density of cancer-associated fibroblasts are directly correlated with poor prognosis in solid tumor^24,25^, by varying the density of fibroblasts in the system and we can first examine the quantitative impact of cell concentration and MMPs in a patho-physiologically relevant setting. Secondly, fibroblasts are highly contractile. To vary the density of fibroblasts allows us to roughly control the mechanical factors in the system through controlling density of fibroblasts. Thirdly, tumor microenvironment in vivo is packed with dense cellular components where intercellular distance is small. By varying density of cell populations in the system we can modulate the average cell–cell distances and better mimic the tumor microenvironment. We further examine the global and local remodeling of the ECM in monoculture and co-culture conditions. Our results show that fibroblasts significantly increase the motions of tumor cells, and this originates from a variety of factors. Fibroblasts compact the ECM and alter matrix architecture. Furthermore, the full impact of fibroblasts is dependent on MMPs, which not only influence the migration of tumor cells but also the ECM profile.

## Results

**Figure 1:**
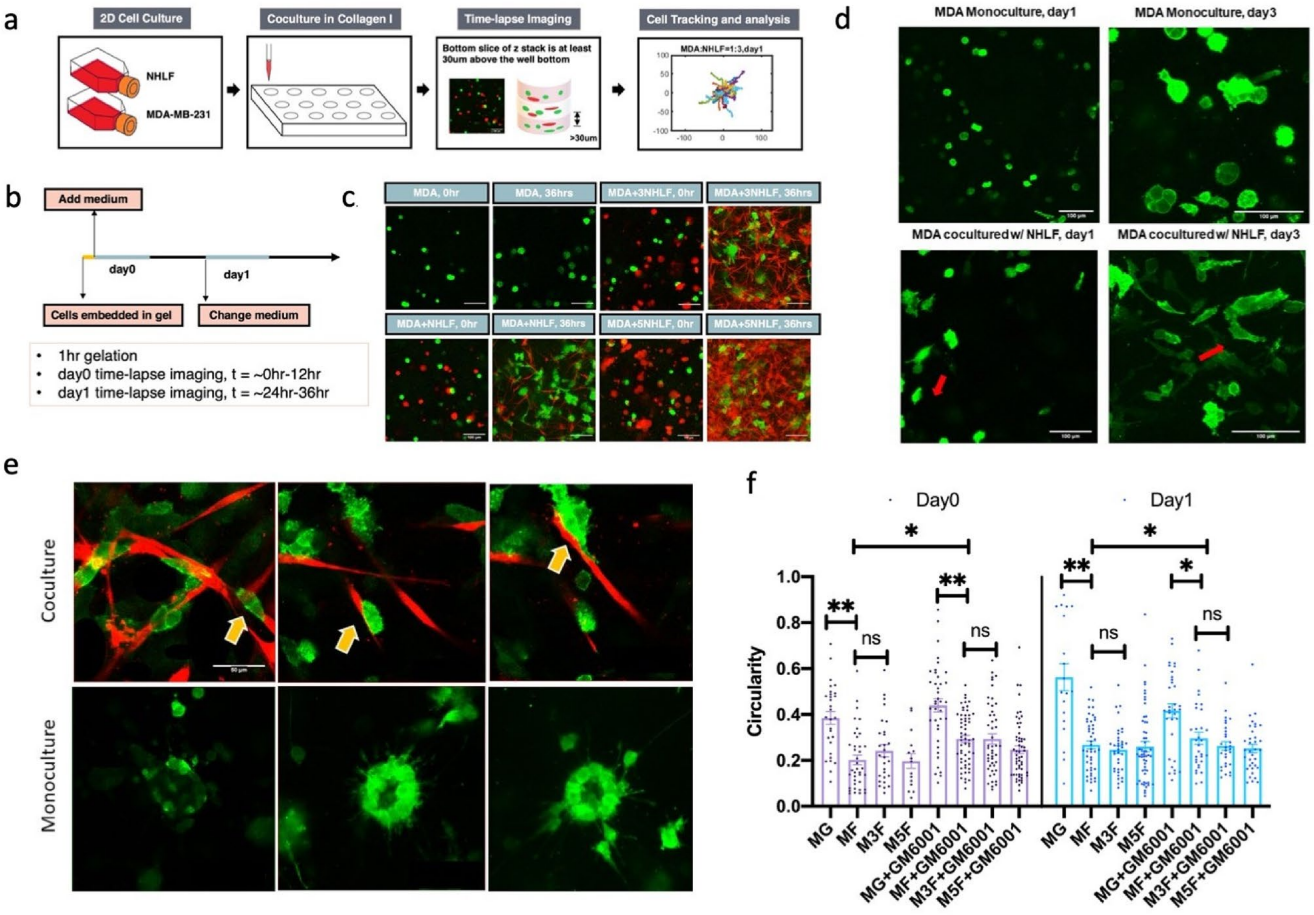
Experiment set-up of the in vitro MDA-MB-231 + NHLF coculture. (**a**) Brief introduction to the pipeline. (**b**) Diagrams show the timeline of the image acquisition. (**c**) Representative images of MDA-MB-231 (shortened as MDA, shown in green channel) and normal human lung fibroblast (shortened as NHLF, shown in red channel). "MDA" indicates MDA-MB-231 monoculture, "MDA + NHLF" indicates co-culture with initial cell concentration ratio between MDA-MB-231 and NHLF of 1:1; "MDA + 3NHLF" indicates co-culture with initial cell concentration ratio between MDA-MB-231 and NHLF of 1:3; "MDA + 5NHLF" indicates co-culture with initial cell concentration ratio between MDA-MB-231 and NHLF of 1:5. The scale bar is 100 µm. (**d**) MDA-MB-231 morphology in long term culture. Scale bar is 100 µm. (**e**) MDA-MB-231 (green) in close contact with NHLF (red) can be seen in co-culture conditions (top row). In monoculture, petal-like structure can be seen after around 4–5 days post cell embedment. Scale bar is 50 µm. (**f**) Circularity of MDA-MB-231 cells on day0 and day1. For MDA-MB-231 monoculture, at least 15 cells were traced and for cocultures, around 30 cells traced. Data were collected from two independent experiments with four replicates. One way ANOVA with post-hoc Tukey HSD Test was performed. *p < 0.05, **p < 0.01.

We generate monoculture and co-culture systems in 3D collagen I matrices, and we perform time-lapse imaging and image analysis (Fig. 1a–c). Under monoculture conditions (tumor cells only), we find that cells appear to be in a more rounded morphology, whereas in co-culture conditions, tumor cells acquire a more extended morphology (Fig. 1d,f and SI Video 1). Circularity of MDA-MB-231s drops significantly in the presence of NHLF regardless of GM6001 treatment. The presence of GM6001 in MF conditions can rescue the decrease of circularity on day0. Significant differences are not detected across co-culture conditions. Close proximity and dense mixtures of tumor cells and fibroblasts can be observed at around day 4 and 5 (see Fig. 1e). In contrast, large round clusters of tumor cells are observed in monoculture conditions.

**Figure 2:**
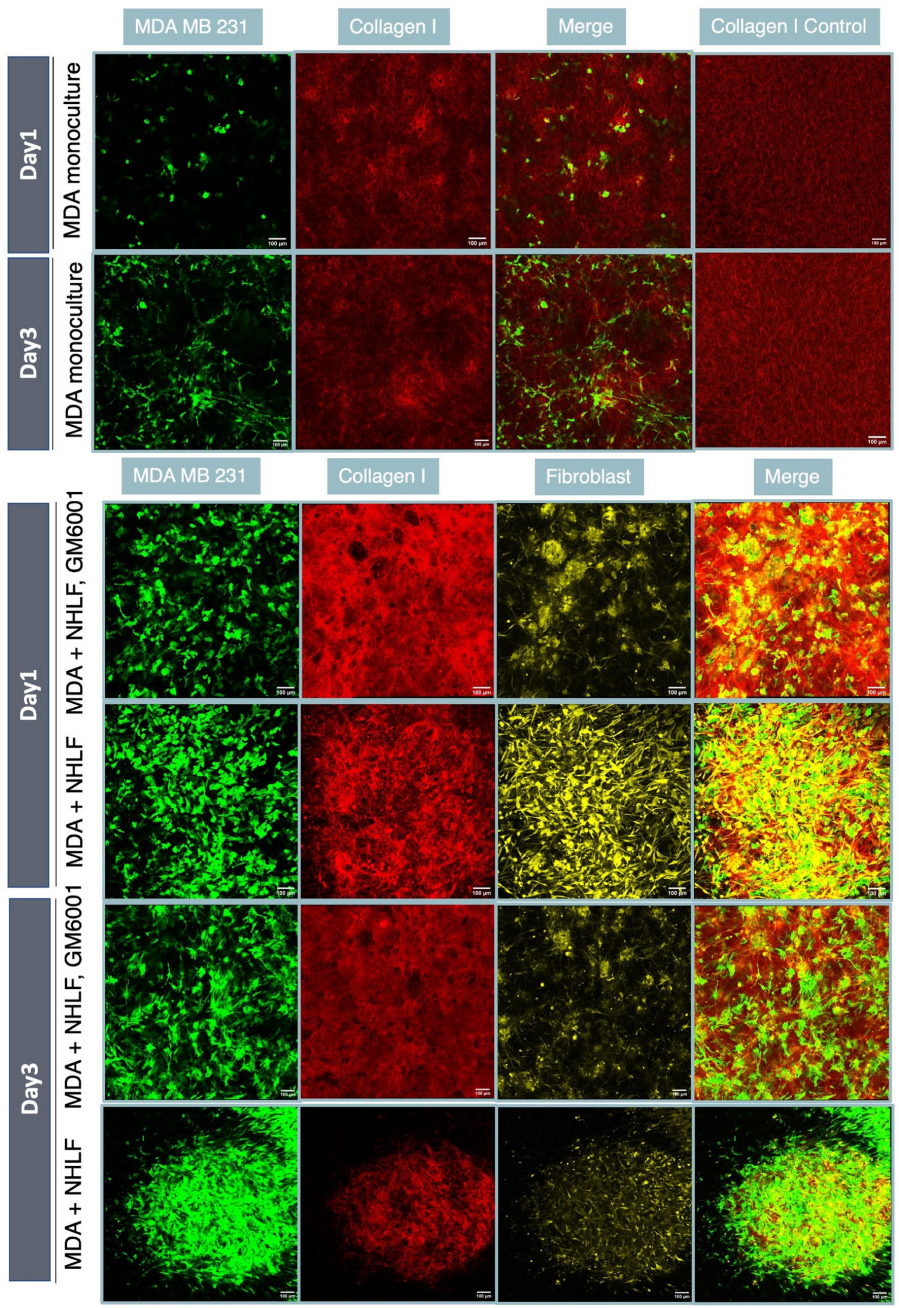
NHLFs contribute the dissemination of MDA-MB-231 through ECM remodeling. Comparison of the spatial environment of the MDA-NHLF coculture on day 1 and day 3, with and without GM6001 treatment (20 µM). In MDA-MB-231 monoculture, collagen bundling or thickening mostly occurred on the peripheral region near MDA cells, as shown in images from row 1 and row 2. On day1 in MDA-NHLF coculture, with 20 µM GM6001 treatment, the collagen gel remained intact and fibroblasts were trapped in the middle of gel. In conditions without GM6001 treatment, fibroblast filled up the whole gel space evenly. On day3, with GM6001 treatment, the collagen gel collapsed to the middle. "MDA + NHLF" indicates co-culture with initial cell concentration ratio between MDA-MB-231 and NHLF of 1:1. Scale bar is 100 µm. Note that fluorescently stained collagen is used here to visualize subtle architectural change. For all other quantification unless otherwise noted, unstained type I rat tail collagen is used.

To track the collagen structure change over time, we collected images of fluorescently stained collagen with cells embedded (see Fig. 2). The stained collagen may have different properties than normal collagen due to the staining process (see “Methods”), which could explain differences in cell morphologies. In monoculture, cell aggregates emerge, whereas in co-culture with fibroblasts, cells tend to occupy more uniformly throughout the matrix (SI Video 2). However, suppressing MMP activity via GM6001 (a pan-MMP inhibitor) leads to more clumped cells, including in the fibroblast population. On day3, collagen that harbors both MDA-MB-231s and NHLFs are more likely to collapse and contract compared with GM6001 treated groups, which highlights the important role of ECM integrity and degradation in regulating cell behaviors in TME.

**Figure 3:**
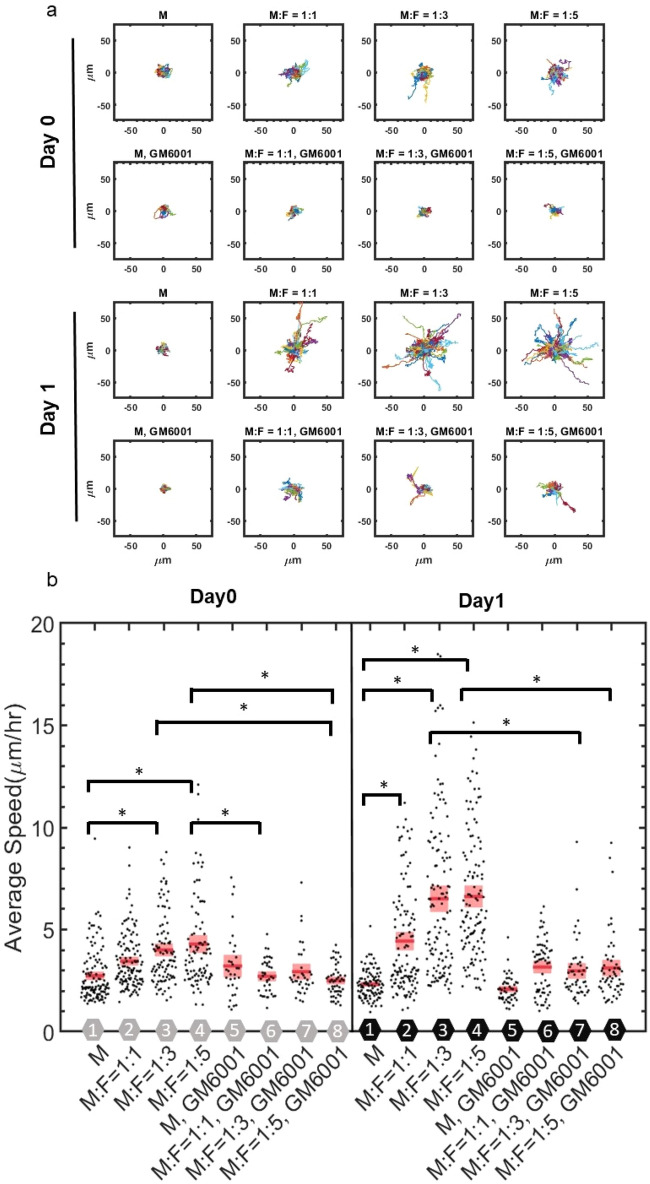
Migration of MDA-MB-231 cells depend on NHLF concentration. (**a**) Overlaid trajectories from each condition truncated at 180 min. M indicates MDA-MB-231, and F indicates NHLF. The ratio indicates the initial seeding concentration ratio of MDA-MB-231 to NHLF cells. GM6001 indicates 20 µm GM6001 treatment. (**b**) Average speed of MDA-MB-231 cells from different conditions. Red line indicates the mean value and pink boxes indicate the 95% confidence interval. Each condition is number coded as indicated by the hexagon along x axis. Gray hexagons indicate day0, black hexagons indicate day1, GM6001 indicates 20 µm GM6001 treatment. In each condition, around 50–120 cells were tracked. Data were collected from at least 3 independent experiments with 2 replicates sampled in each experiment. One way ANOVA was performed to show the difference across all conditions. Tukey’s honest significant difference criterion is used in post-hoc analysis. Histogram data are shown in SI Fig. 2.

We track the migration of tumor cells in the 3D ECM with varying concentrations of fibroblasts on day 0 and day 1 after cell seeding. We find that the trajectories and speeds are larger for co-culture conditions (Fig. 3a,b). Moreover, this is more apparent on day1 after cell seeding. In monoculture conditions, trajectories and speeds are relatively small even after 1 day in culture. When MDA-MB-231 cells are cultured with equal amount of MDA-MB-231 by themselves, there is no significant difference in MDA-MB-231 migratory behaviors (SI Fig. 1). These results indicate time-dependent evolution of the fibroblast-mediated microenvironment toward a state that facilitates tumor migration. Moreover, increased cancer cell invasiveness is dependent on fibroblast concentration. More fibroblasts lead to faster cancer cells, but this effect plateaus at relatively high fibroblast concentrations, suggesting saturation of invasion-promoting signals generated by fibroblasts and/or high consumption rate of nutrients due to high cell density, as there is limited nutrient supply in the cell culture well. Alternatively, the limited medium supply of the culture chamber may be another contributing factor to the plateaued effect.

**Figure 4:**
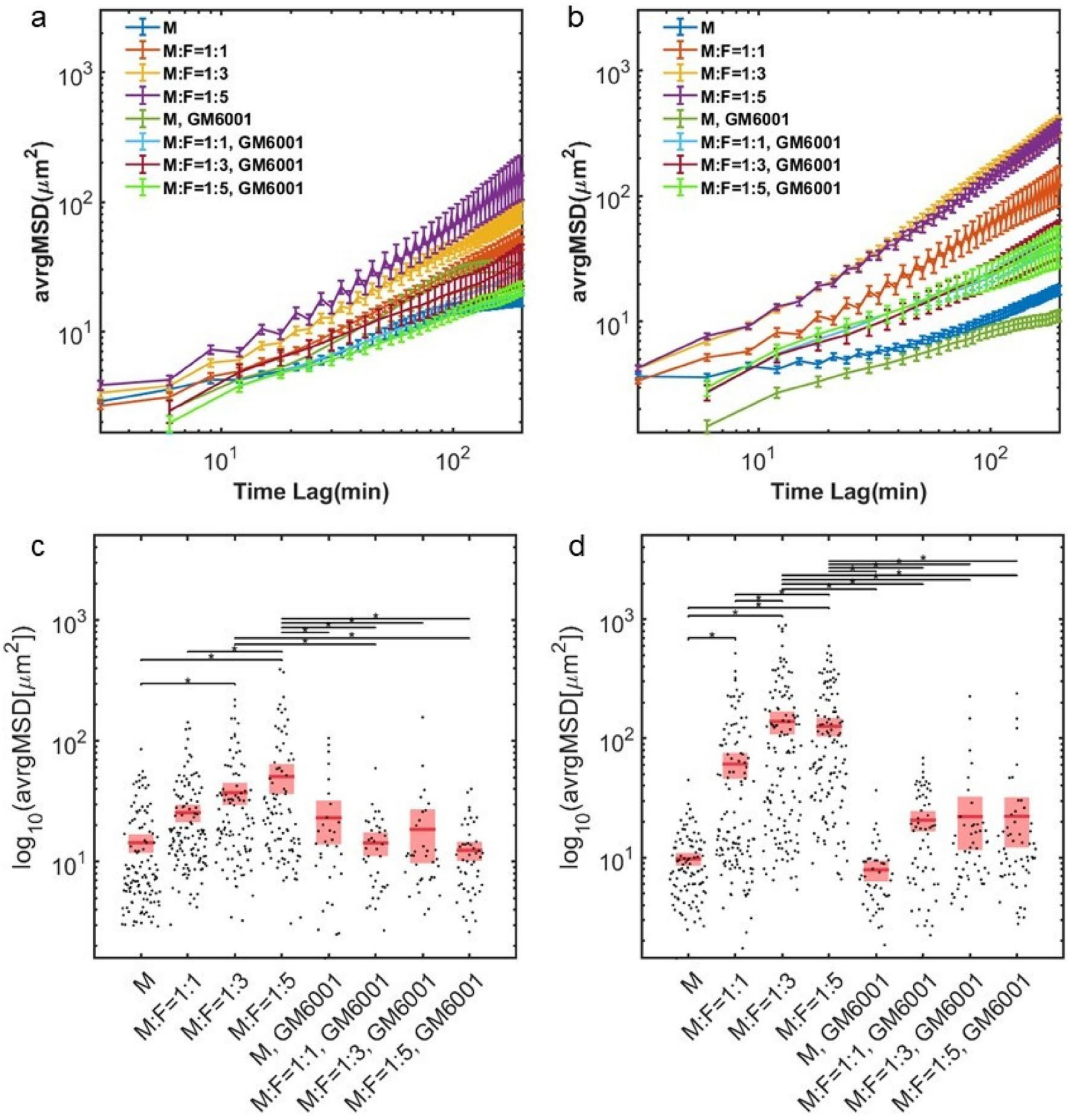
MSD profiles of MDA-MB-231 cells are dependent on NHLFs. (**a**) Average MSD on day0, error bar in SEM. (**b**) Average MSD on day1, error bar in SEM. (**c**) MSD taken at the 90 min time lag, day 0. (**d**) MSD taken at the 90 min time lag, day1. M indicates MDA-MB-231 and F indicates NHLF. The ratio indicates the initial seeding concentration ratio of MDA-MB-231 to NHLF cells. Red line indicates the mean value and pink boxes indicate the 95% confidence interval. One way ANOVA was performed to show the difference across all conditions. * indicates the two compared conditions are significantly different (*p* < 0.05). Histogram data are shown in SI Fig. 3. In each condition, around 50–120 cells were tracked. Data were collected from at least 3 independent experiments with 2 replicates sampled in each experiment.

**Figure 5:**
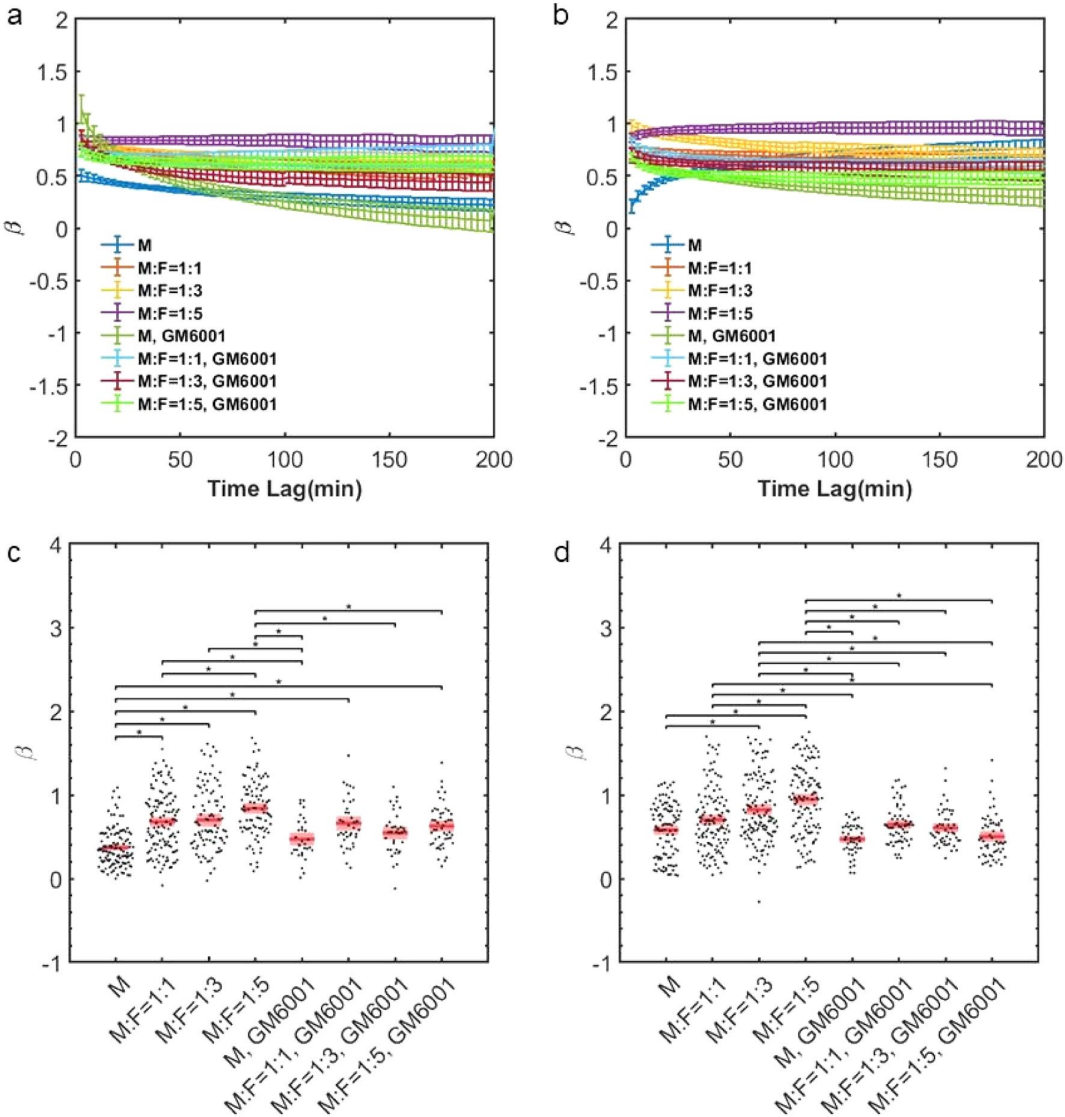
MDA-MB-231s migrate with altered diffusivity in the presence of NHLFs. (**a**) Average beta on day0, error bar in SEM. (**b**) Average *β* on day1, error bar in SEM. (**c**) *β* taken at the 90 min time interval, day 0. (**d**) *β* taken at the 90 min time interval, day 1. M indicates MDA-MB-231, and F indicates NHLF. The ratio indicates the initial seeding concentration ratio of MDA-MB-231 to NHLF cells. Red line indicates the mean value and pink boxes indicate the 95% confidence interval. One way ANOVA was performed to show the difference across all conditions. * indicates the two compared conditions are significantly different (*p* < 0.05). Histogram data are shown in supplementary information SI Fig. 4. In each condition, around 50–120 cells were tracked. Data were collected from at least 3 independent experiments with 2 replicates sampled in each experiment.

We further investigate the statistical properties of the tumor cell motions. By considering tumor cell mean-squared displacements (MSDs), we find that fibroblasts increase their diffusivity. The magnitude of the MSDs is larger in co-culture conditions in a fibroblast concentration and time dependent manner (Fig. 4). Additionally, *β*, the logarithmic derivative of the MSD, is higher in co-culture conditions (Fig. 5). β provides insights toward the physical nature of the cell motions.  β= 0 indicates confined motion, characteristic of cells that are caged, and β = 1 indicates diffusive (unconfined and random) motion. Co-culture conditions enable tumor cells to migrate in a more diffusive manner.

Furthermore, the influence of fibroblasts on tumor migration is dependent on MMPs. Inhibiting MMPs with GM6001 reduces the fibroblast-mediated increases in tumor cell speed, MSD, and β (Figs. 3, 4, 5) However, the co-culture effects are not completely abolished through MMP-inhibition, as shown by the comparisons with tumor cell monocultures, suggesting additional biophysical influences by fibroblasts. Histograms of average speed, average MSD and average β are shown in SI Figs. 2, 3 and 4 respectively.

**Figure 6:**
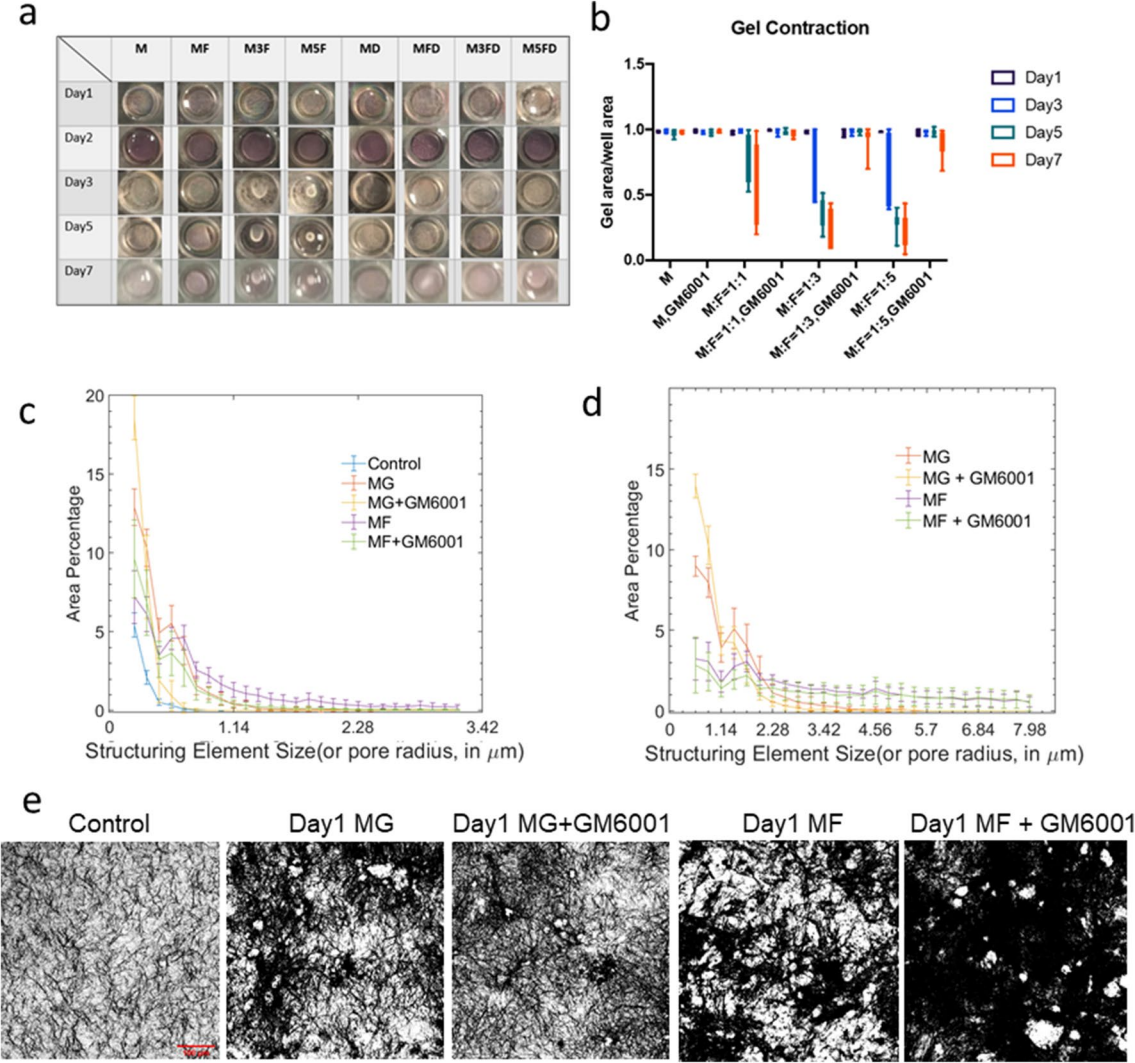
Quantification of global and local changes in collagen scaffold. (**a**) Gel compaction recorded longitudinally from day1 to day7."M" indicates MDA-MB-231 monoculture, "MF" indicates initial seeding concentration between MDA-MB-231 to NHLF is 1:1; "M3F" indicates initial seeding concentration between MDA-MB-231 to NHLF is 1:3; "M5F" indicates the initial seeding concentration between MDA-MB-231 to NHLF is 1:5; "MD" indicates MDA-MB-231 monoculture with 20µM GM6001 treatment; "MFD" indicates an initial seeding concentration ratio of 1:1 between MDA-MB-231 to NHLF with 20µM GM6001 treatment; "M3FD" indicates an initial seeding concentration ratio of 1:3 between MDA-MB-231 to NHLF with 20µM GM6001 treatment; "M5FD" indicates an initial seeding concentration ratio of 1:5 between MDA-MB-231 to NHLF with 20µM GM6001 treatment. (**b**) Quantification of gel compaction. The ratio indicates an initial seeding concentration between MDA to NHLF in each condition. Data from at least triplicates are averaged, and in each repeat there are duplicate wells for each condition. Error bars in SEM. (**c**) and (**d**) Pore size distribution on day 0 (**c**) and day 1 (**d**). Around 5–12 images (around 1000×1000 pixels) from two replicates were used for the quantification. The existence of NHLF contributes to the erosion of collagen scaffold and lead to larger hole size overall. GM6001 inhibits ECM erosion and breakage, consistent with the global gel compaction trend. (**e**) Binarized collagen images before pore size measurement. Similar to control conditions, day1 MDA monoculture generally has more small pores visually than coculture conditions, consistent with quantification shown in (**d**).

We next examine the influence of the cells on the global and local ECM behavior. We find that in co-culture conditions, the matrix is globally compacted over time, as shown by the decrease in whole gel radius (Fig. 6a,b). This compaction is mediated by fibroblasts, as monocultured tumor cells did not significantly shrink the gel radius. Moreover, this compaction process is dependent on MMPs, as MMP inhibition abrogates the compaction of the gel even in co-culture conditions with relatively high fibroblast concentrations (SI Video 3). As fibroblasts are highly contractile and pull collagen dynamically, we tracked fluorescent debris in our models to quantify gel contraction from day0 to day1 and to differentiate cell active migration from passive movement due to local gel contractions by neighboring cells (SI Fig. 5). Our data has shown that gel contraction on day0 is similar with MDA-MB-231 migration on day0 which suggest that cell motility is largely dependent on gel dynamic deformation in this time frame. However, MDA-MB-231s demonstrate much increased motions on day1 compared with gel contraction, which suggest active migration in addition to gel contraction (SI Video 4). When we examined local ECM architecture under high resolution confocal microscopy with collagen fibers labeled fluorescently, we find that the architecture of the ECM is drastically different in the presence of fibroblasts, in which rampant remodeling occurs leading increased open spaces (2). We also quantified pore size with a morphological opening method (see “Methods”) as demonstrated by SI Fig. 6. Our quantification (Fig. 6c,d) of pore size distribution across all conditions shows that the presence of NHLF in the culture appears to contributes to larger pores or empty space. Control group (without cells) have smallest pore sizes based on our metric. MG or MG + GM6001 groups on both day0 and day1 have more large holes compared with control group. Hole sizes are generally larger (higher plateau on the right end of axis, Fig. 6c,d) in MF groups. MF conditions have higher number of pores of radius between 1 and 5 µm. However, for larger pores (radius larger than 5 µm), it is hard to differentiate MF and MF + GM6001. This may partially come from the pseudo-pores from cells’ occupation in coculture conditions. Additionally, caveats must be taken to interpret hole size distribution in coculture conditions as elaborated by SI Fig. 7a, When cell density is high, the holes in the collagen channel of our images are also caused by the presence of cells occupying the space as shown by SI Fig. 7b. And in matrices with high cell density, as cells are occupying most of the space, it is difficult to assess pore sizes based on regions without cells.

**Figure 7:**
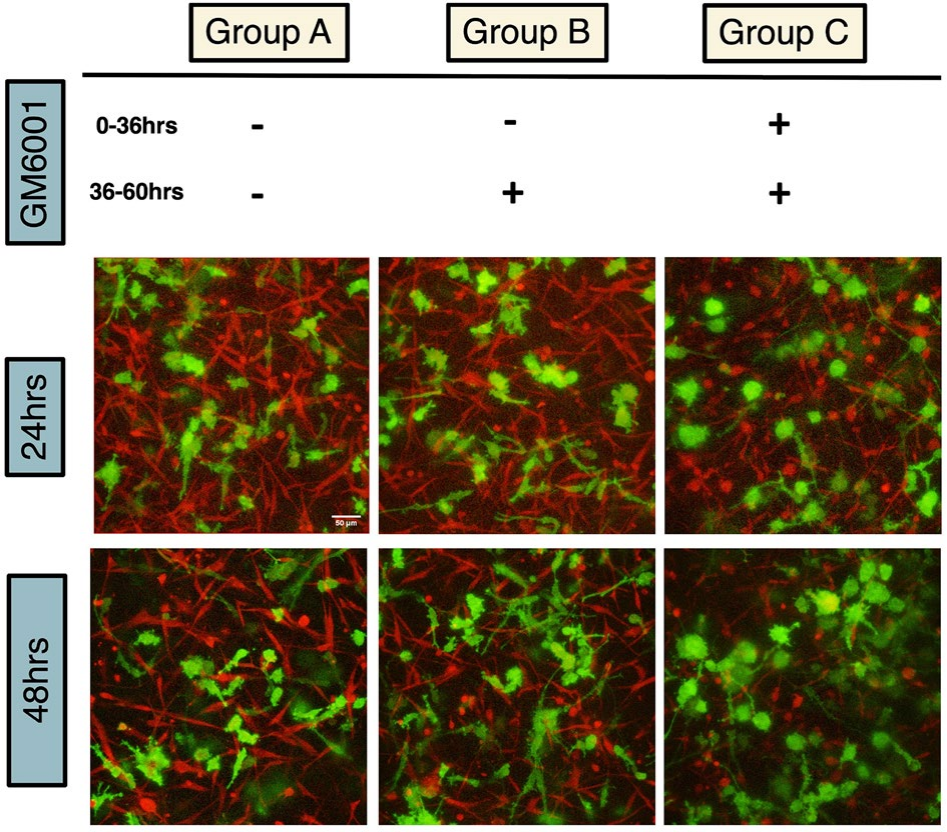
Addition of GM6001 into MDA-NHLF coculture system after day 1 did not inhibit MDA migration compared with GM6001 addition since cell embedding, indicating the mechanism of GM6001 inhibition is through inhibiting collagen remodeling induced by NHLF. In Group A, GM6001 was not present throughout the experiment. In Group B, GM6001 was added at 36 h after gelation. In Group C, GM6001 was added at 0 h during cell embedding. The bottom images show morphology of MDA-MB-231 cells at 24 h and 48 h after gelation. The initial seeding density of MDA-MB-231 is 800 K/ml and NHLF is 2400 K/ml. Scale bar is 50 µm.

Finally, we investigate whether delayed MMP-inhibition is effective in suppressing tumor cell invasion. We find that if we do not treat co-culture samples with GM6001 on days 0 and 1, thus allowing 2 days for ECM remodeling, and start treatment on day 2, tumor cell motions are not impacted by the drug (Fig. 7). This indicates that the ECM is already remodeled sufficiently over 2 days to achieve maximal impact on tumor cell migration. Co-culture samples thus require continuous treatment from initial stages in order to suppress invasion-promoting remodeling of the nominal ECM. The influence of the initial remodeling appear persistent after MMPs are inhibited subsequently.

## Discussion

In this study, we demonstrate that fibroblasts mediate increased motility in tumor cells. While this process can be modulated by many factors in the cascade of fibroblast-ECM-tumor cell interactions, our results indicate that MMPs are a major, but not complete, contributing factor. Prior studies have shown that adhesions between tumor cells and fibroblasts are important for fibroblast-mediated invasion^12^. Additionally, fibroblasts can secrete paracrine signaling factors and ECM proteins such as fibronectin, which can increase tumor cell motility^26,27^. Here, we show that, in dense 3D collagen matrices, these effects may be subsequent and potentially synergistic to the initial impact of MMP-dependent matrix remodeling. Thus, the temporal sequence of stroma-mediated effects is important to consider.

Moreover, in our images, we find that while at times tumor cells are in contact with fibroblasts during their motions, this is not true for all cases. As shown in SI Video 1, Tumor cells in fibroblast-remodeled microenvironments can move rapidly even when not in direct contact with the fibroblasts themselves, suggesting that the remodeled ECM contains cues that are major factors that drive invasiveness. Our findings highlight the heterotypic microenvironment as a dynamically evolving space in which stromal cells remodel the ECM and new local cues are progressively being manufactured.

From our MSD results, we find that tumor cells typically migrate in a sub-diffusive manner in 3D ECMs, indicative of confinement. Physically, this is likely due to the dense ECM inducing a caging effect. However, in co-culture conditions, the migration becomes closer to being diffusive (β being closer to 1), particularly with high concentrations of fibroblasts. This suggests that fibroblasts can serve the function of uncaging tumor cells. MMPs are a key mediator of this process. Interestingly, however, even with MMPs inhibited, the presence of fibroblasts can still increase tumor cell movements. This suggests additional factors are involved. In particular, contractile forces from fibroblasts can remodel the ECM, generating alignment cues^15^, non-linear stiffening^14,28^, and viscoplastic reorganization^6–8^—factors that can contribute to tumor invasion^8,16–18^.

MMPs have been extensively studied, and tumor cells can utilize them to degrade the ECM to facilitate migration^3^. In dense ECMs with pore sizes smaller than the nucleus, MMPs are required for migration^2^. Here, we show that fibroblasts, which are prominent in the solid tumor microenvironment and in many tissues commonly associated with metastatic sites (e.g. lung, liver), can significantly enhance MMP-mediated cancer cell motility. This further implicates the important role of heterotypic microenvironments and cooperative effects in facilitating tumor invasion. Our results suggest that tissues rich in stromal cells and ECM may already be sufficiently conducive to tumor migration even without ECM remodeling by the tumor cells themselves, and that this process may not depend on specialized cancer-associated fibroblasts, as we used normal human lung fibroblasts in this study.

Furthermore, our findings implicate MMPs in the compaction of the ECM. MMPs are traditionally known for their role in matrix degradation. Their potential roles in the mechanical remodeling of the ECM are not well understood. Mechanical remodeling—ECM deformation and formation of topographical cues—are typically associated with actomyosin and tension mediated processes. Our results show that at the global scale, MMPs mediate the compaction of the whole gel (Fig. 6), and at the local scale, MMPs facilitate tearing of the matrix and formation of gaps (Fig. 2). Previous work has shown that mechanical forces generated by cells, particularly dynamic forces, can be used to recruit ECM toward cells in a mechanically irreversible manner, as ECM bonds can break under tension^6,9^. Our findings suggests that MMPs can facilitate this process, contributing to ECM compaction and tearing by locally weakening the ECM and making it more susceptible for mechanical remodeling.

Finally, the reliance of tumor invasion on MMPs in dense ECMs is time-dependent, as inhibition of MMPs after 2 days of co-culture without initial inhibition does not reduce tumor cell migration. This indicates that on the time-scale of hours to days, the ECM can be irreversibly remodeled by stromal cells into an invasion promoting microenvironment. Thus, MMP-inhibition treatments may only be effective at the very early stages of tumor progression or at the earliest time points when tumor cells reach secondary sites. This also suggests that pre-emptive MMP-inhibition may suppress new sites from promoting further tumor invasion.

## Conclusions

The dense ECM can mechanically restrict cell invasion. Stromal cells, particularly fibroblasts, can facilitate tumor invasion via both MMP dependent and independent mechanisms. In the presence of fibroblasts, the microenvironment is dynamic, evolving toward an invasion-promoting state that is highly distinct from that of the nominal reconstituted ECM in vitro. New local ECM architectures emerge, with features that are conducive to cell invasion. MMPs contribute to the microenvironment remodeling process, not only through matrix degradation but also through facilitating local and global ECM mechanical reorganization. Overall, our results support that modulating regulators of ECM remodeling, particularly stromal cell-mediated activities, is critical toward controlling tumor invasion.

## Methods

### Cell culture

MDA-MB-231 cells transfected with Lifeact GFP were a gift from the Lauffenburger lab. They were cultured in DMEM with 10% fetal bovine serum (FBS) and 1% Pen-Strep. Fibroblasts were primary normal human lung fibroblasts (ATCC, PCS-201-013), and they were cultured in Lonza FGM-2 BulletKit(CC-3132) or RPMI with 10% FBS. Cells were all incubated at 37 °C and 5% CO_2_.

### 3D tissue culture experiments

Cells were seeded into 2 mg/mL rat tail type I collagen gels (Corning). Briefly, acid solubilized collagen I was neutralized with NaOH and mixed with cells on ice, followed by gelation at 37 °C. Cells were seeded at varying concentrations, with 1× indicating 800 K cells/mL. Fibroblasts were mixed at 0X to 5X the concentration of cancer cells. For drug studies, GM6001 (20 µM) was mixed into the collagen gel solution and into the media. For labeled ECM studies (Fig. 2), the initial collagen solution was labeled with Alexa Fluor 647 NHS Ester (Succinimidyl Ester) and dialyzed as before^6^. Note that only Fig. 2 used stained collagen. In all other quantification, unless other noted, unstained type I collagen is used. Finally, we coated the surface of the multi-well plates used for imaging with polydopamine to anchor the collagen gel^29,30^.

### Imaging

Imaging was performed using a Leica SP8 confocal microscope. For migration studies, we used a 20× 0.75 NA objective.

### Analysis

Cell circularity was measured on ImageJ with the analyze particle function. For cell motions, cells were tracked manually or with TrackMate^31^ on Image J. Each cell center was calculated by averaging coordinates of corresponding traced cell boundary. Average speed of each cell was calculated as the mean of the absolute value of the net displacement of the cell center over 1 h time intervals. Mean squared displacements were computed with the following equation^32^:

1 $$MSD(n) = \frac{1}{N - n + 1}\sum\limits_{i = 0}^{N - n} {\left[ {\left( {x_{i + n} - x_{i} } \right)^2 + \left( {y_{i + n} - y_{i} } \right)^2} \right]}$$

where N indicates the total step number, n indicates the nth step, x is the x-coordinate and y is the y-coordinate. *β* was computed by first locally smoothening the MSD profile with a Guassian-weighted polynomial and then taking the logarithmic derivative of the smoothened MSD over logarithmic time^33,34^, i.e.

2 $$\beta = \frac{{d\left( {ln\left( {MSD} \right)} \right)}}{{d\left( {ln(t)} \right)}}$$

Data points of *b* that are less than zero are considered as noise and discarded. Note that for trajectories-related calculations, 2D trajectories of z-projected cell migration time lapse data were used. ANOVA with Tukey post hoc test was performed for statistical comparisons. * indicates *p* values < 0.05. For quantifying ECM deformation dynamics due to local cell contractions, small fluorescent debris are manually selected for tracking via the following criteria: (1) Debris are well adhered to the matrix instead of to the cell body and are not freely diffusing through the matrix; (2) Neighboring debris show similar movement trend and are moving along gel contraction direction through visual confirmation; (3) Debris appear in more than 80% of the time frames. The trajectories of debris movement and speed/MSD calculation are calculated in the same way as for cell motions. For pore size measurements, we used the sequential morphological opening method with a disk shape as the incremental structuring element as in previous work (see SI Fig. 6a)^35^. The area change from opening is used to approximate the distribution of pores of specific size. It is important to note that in our experiments, cell density is relatively high. Therefore, in monoculture conditions, we did downsampling by cropping regions without cells into 300 × 300 pixels (See SI Fig. 6b). For coculture conditions where cells are occupying most of the gel, we select collagen channels only (see SI Fig. 7).

## Supplementary Information

Supplementary Video 1.

Supplementary Video 2.

Supplementary Video 3.

Supplementary Video 4.

Supplementary Information 1.
