# Supplementary material for: Fibroblast-mediated uncaging of cancer cells and dynamic evolution of the physical microenvironment
Source: Sci Rep. 2022 Jan 17;12:791. doi: 10.1038/s41598-021-03134-w (PMC8764094; doi:10.1038/s41598-021-03134-w)
Supplement: Supplementary file 5 — Supplementary Information 1. [file 41598_2021_3134_MOESM5_ESM.pdf]

1 Supplementary Information

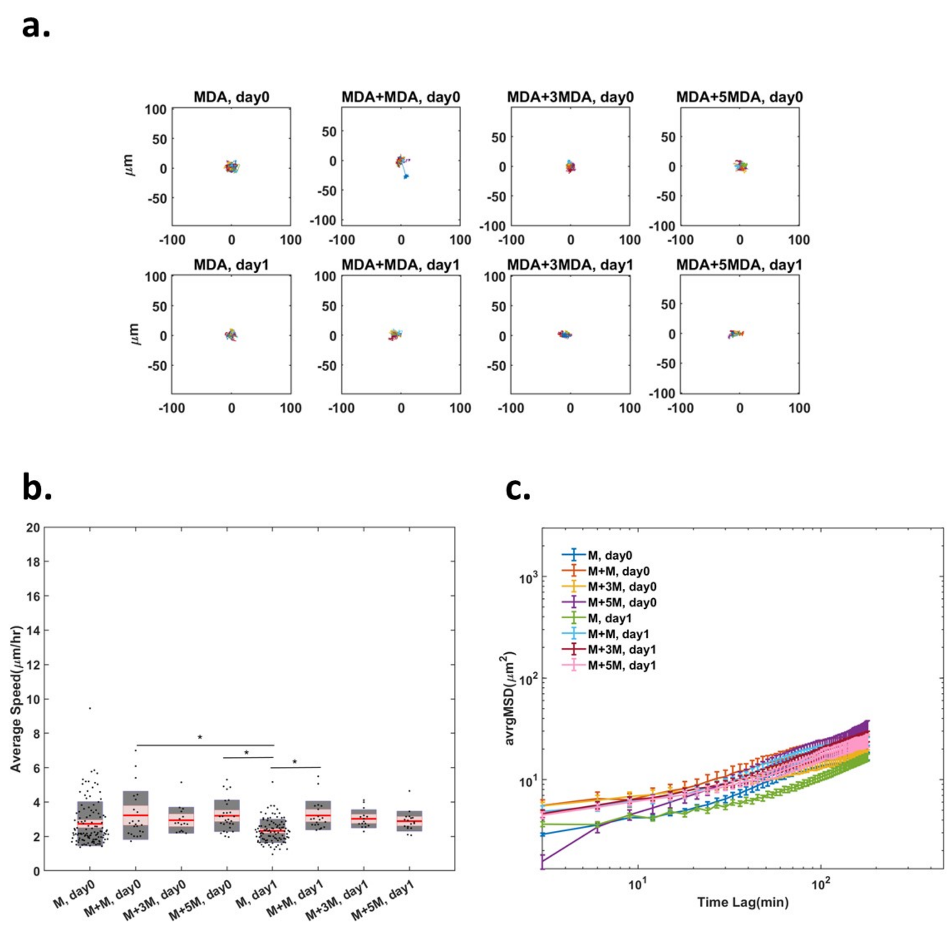

SI Fig 1. MDA-MB-231 migration in monoculture.When NHLFs are replaced with MDA-MB-231 cells, differences in cell concentration in monoculture conditions appear to have low impact on cell migration, relative to coculture conditions. a: Overlaid trajectories truncated at 180min; "MDA" indicate 1X MDA-MB-231 monoculture, "MDA+MDA", "MDA+3MDA", "MDA+5MDA" indicate 2X, 4X and 6X MDA-MB-231 monoculture respectively. b: Average speed, "MDA" is shortened as "M" for brevity. Raw data points are overlaid with gray boxes which indicate +/- one standard deviation and pink boxes which indicate the 95% confidence interval; One way ANOVA was performed to show the difference across all conditions. $*$ indicates the two compared conditions are significantly different ($p < 0.05$). Tukey's honest significant difference criterion is used in post-hoc analysis. c: Average MSD , errorbar in SEM.

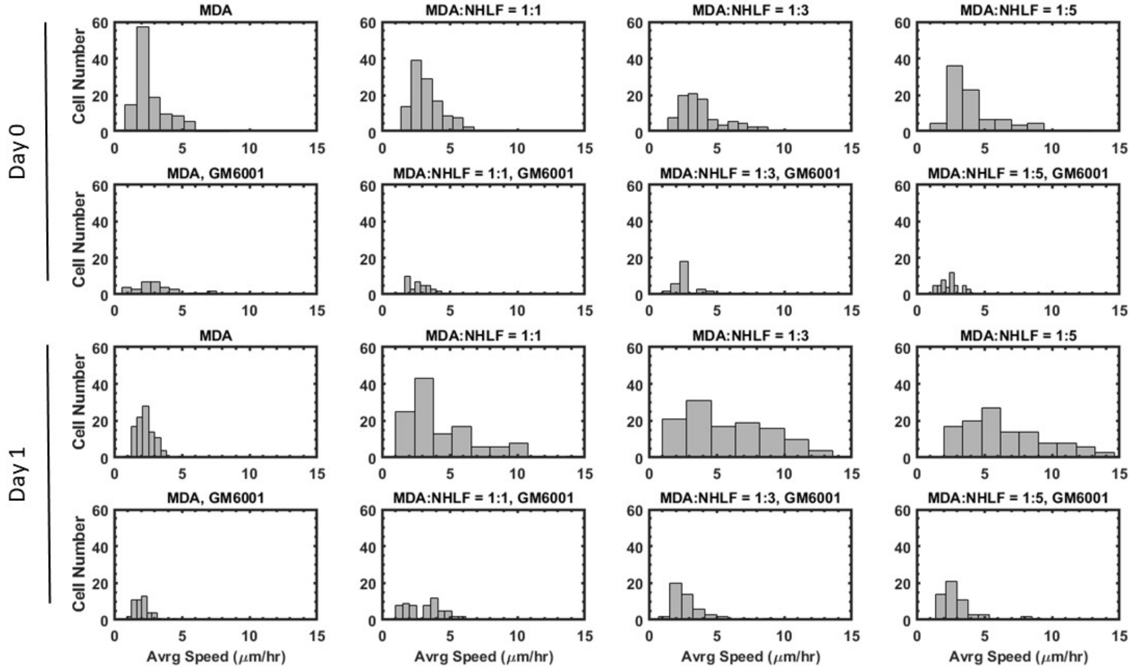

SI Fig 2. Histogram of Average Cell Speed, X axis: average speed. Y axis: number of cells.

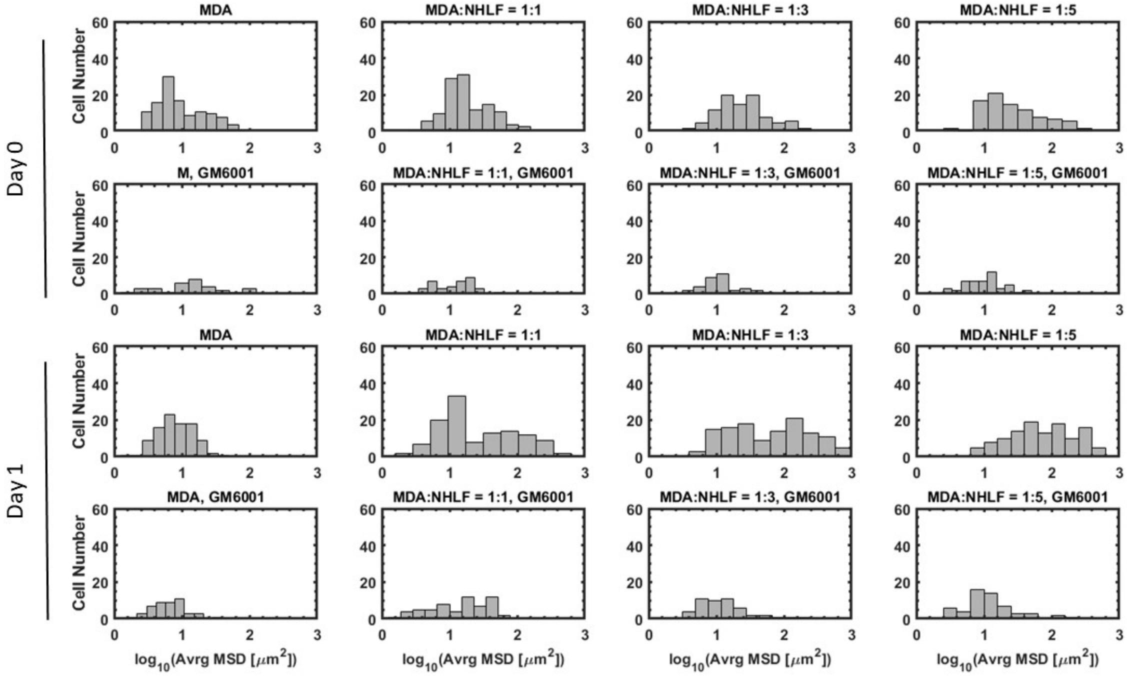

SI Fig 3. Histogram of $\log_{10}(averageMSD)$, X axis: $\log_{10}(averageMSD)$. Y axis: number of cells.

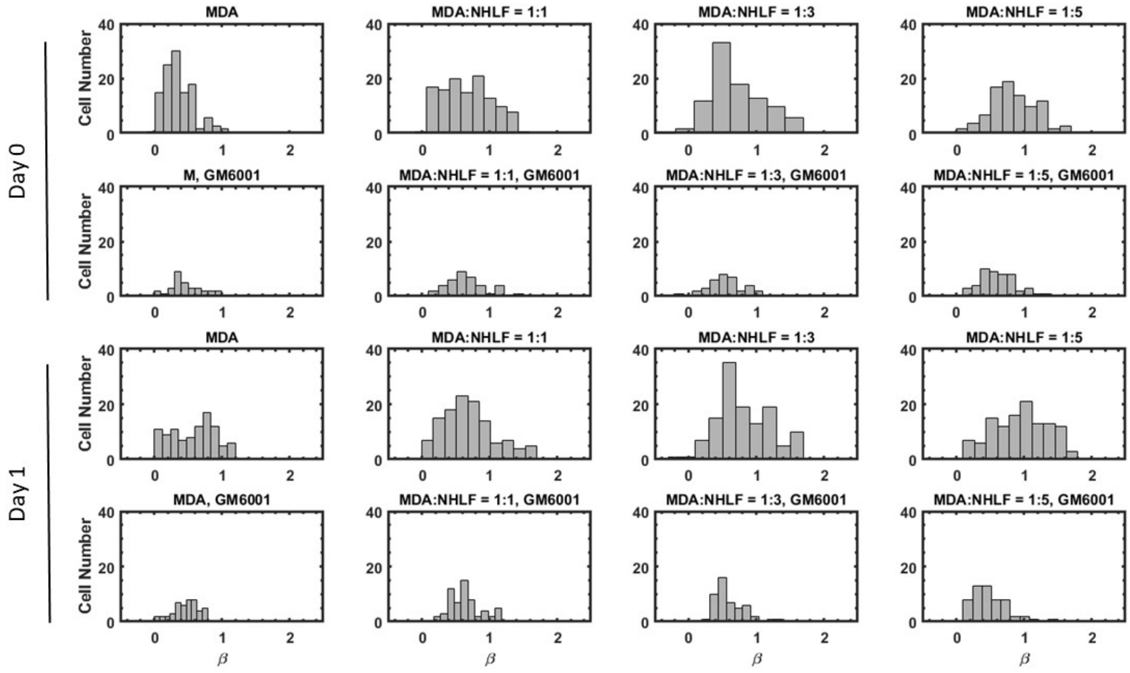

SI Fig 4. Histogram of β, X axis: β. Y axis: number of cells.

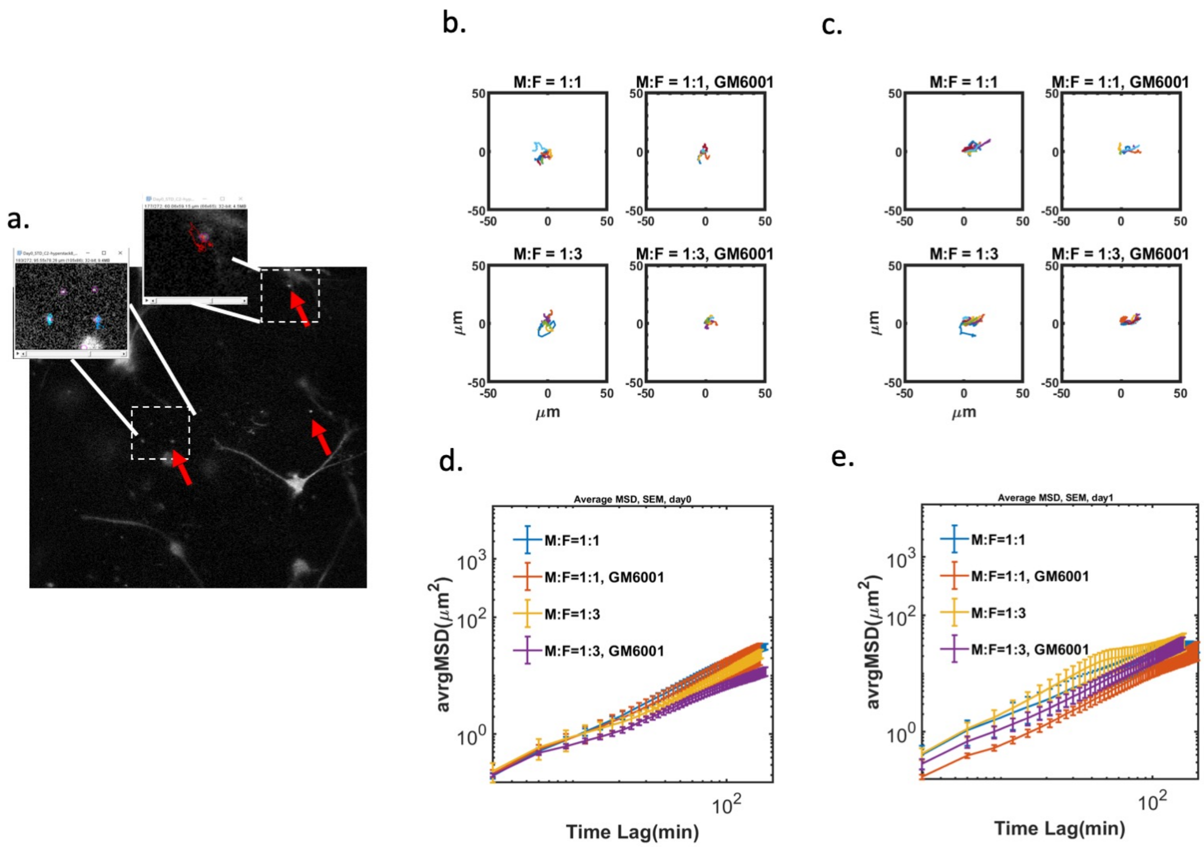

SI Fig 5. Tracked gel compaction on day0 and day1. a. Fluorescent debris attached on collagen gel were tracked over time to capture the movement of collagen gel as a result of fibroblasts' collagen pulling activity. Gel movement is in similar scale with MDA-MB-231 movement on day0 but much less than MDA-MB-231's migration in coculture conditions on day1, suggesting that additional mechanism should contribute to the increased migration of MDA-MB-231 on day1. b&c, trajectories of fluorescent debris on day0(b) and day1(c). d&e, MSD of fluorescent debris on day0(b) and day1(c). Gel contraction happens both on day0 and day1, GM6001 slightly inhibit gel compaction on day1. However, MDA-MB-231 demonstrates higher migration persistence (see Fig4)than gel contraction movement.

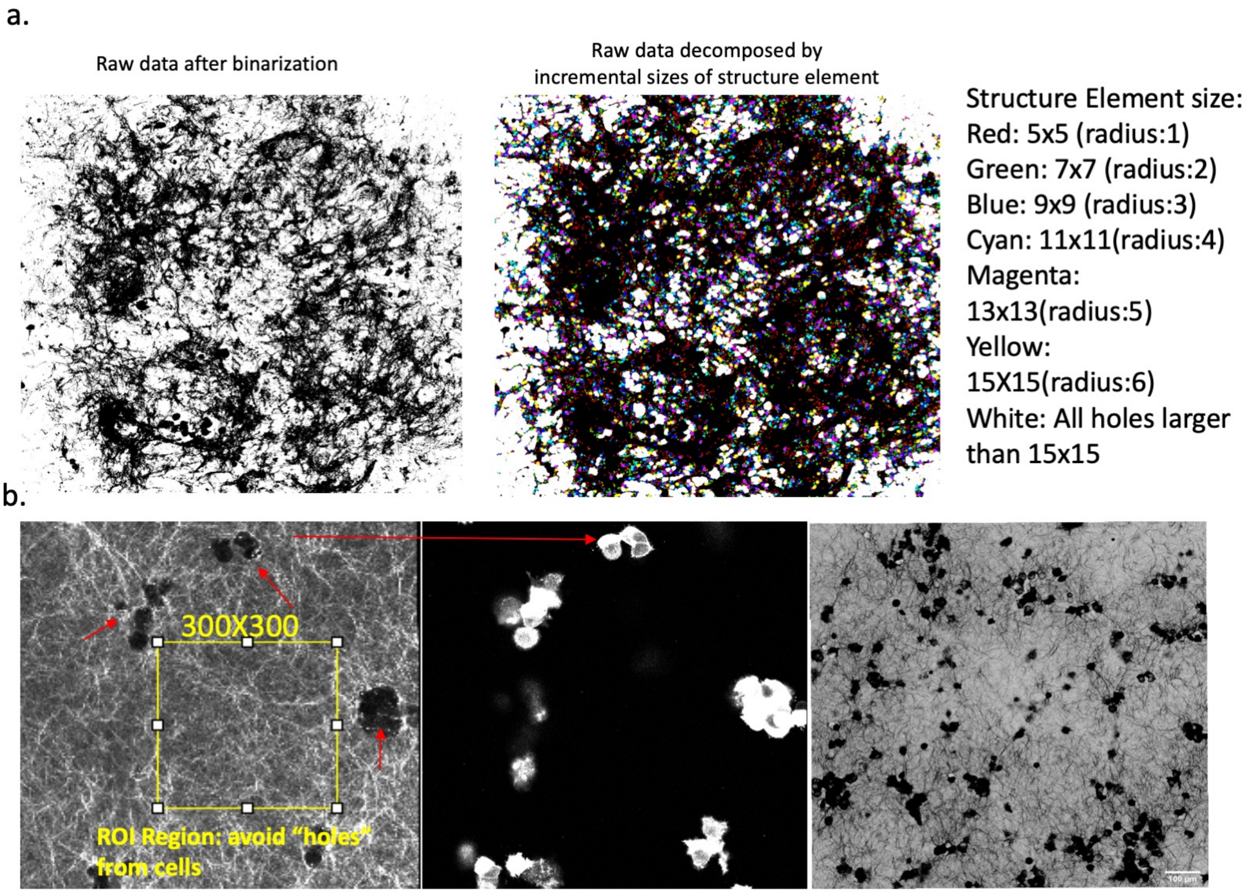

SI Fig 6. Pore size measurement with cells embedded. a. Demonstration of performance of pore size measurement with morphological opening. Pores of different sizes are probed by incremental sizes of kernels as shown by the colorcoded dots. b. In MDA only conditions, images are pre-processed to avoid the confounding effect from cells' occupation(pseudo-holes). Multiple non-cells region are down-sampled(300X300 pixels) and quantified.

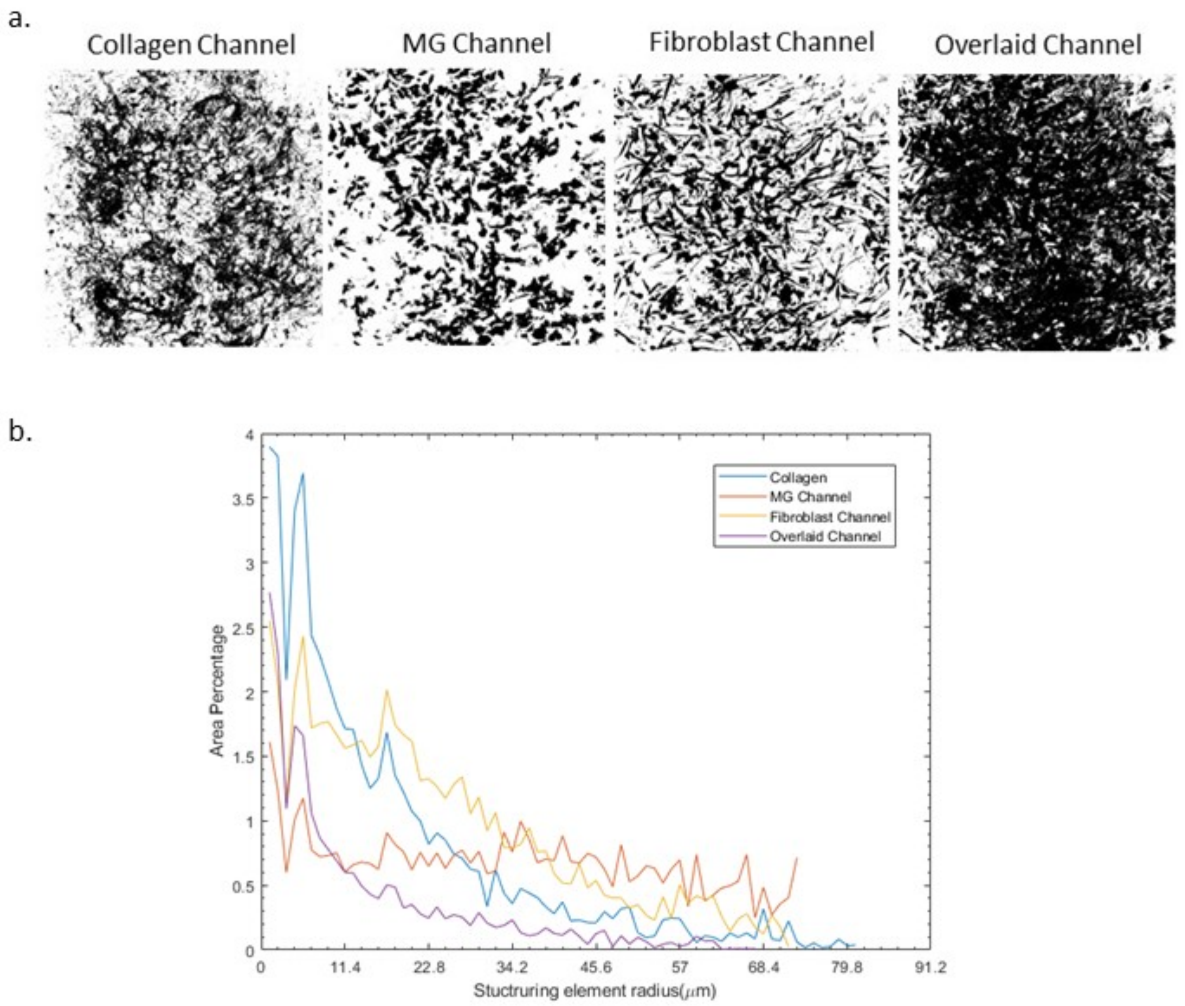

SI Fig 7. Pore size measurement with cells embedded: coculture conditions. Unlike monoculture conditions where total cell number is much smaller, the pore size measurement in coculture conditions are more susceptible to cells' occupation. Caveats must be taken when interpreting the pore size quantification in this scenario. a. Binarized images of each channel in one MDA+NHLF coculture example data set. b. Quantification of the size distribution of holes in each channel.

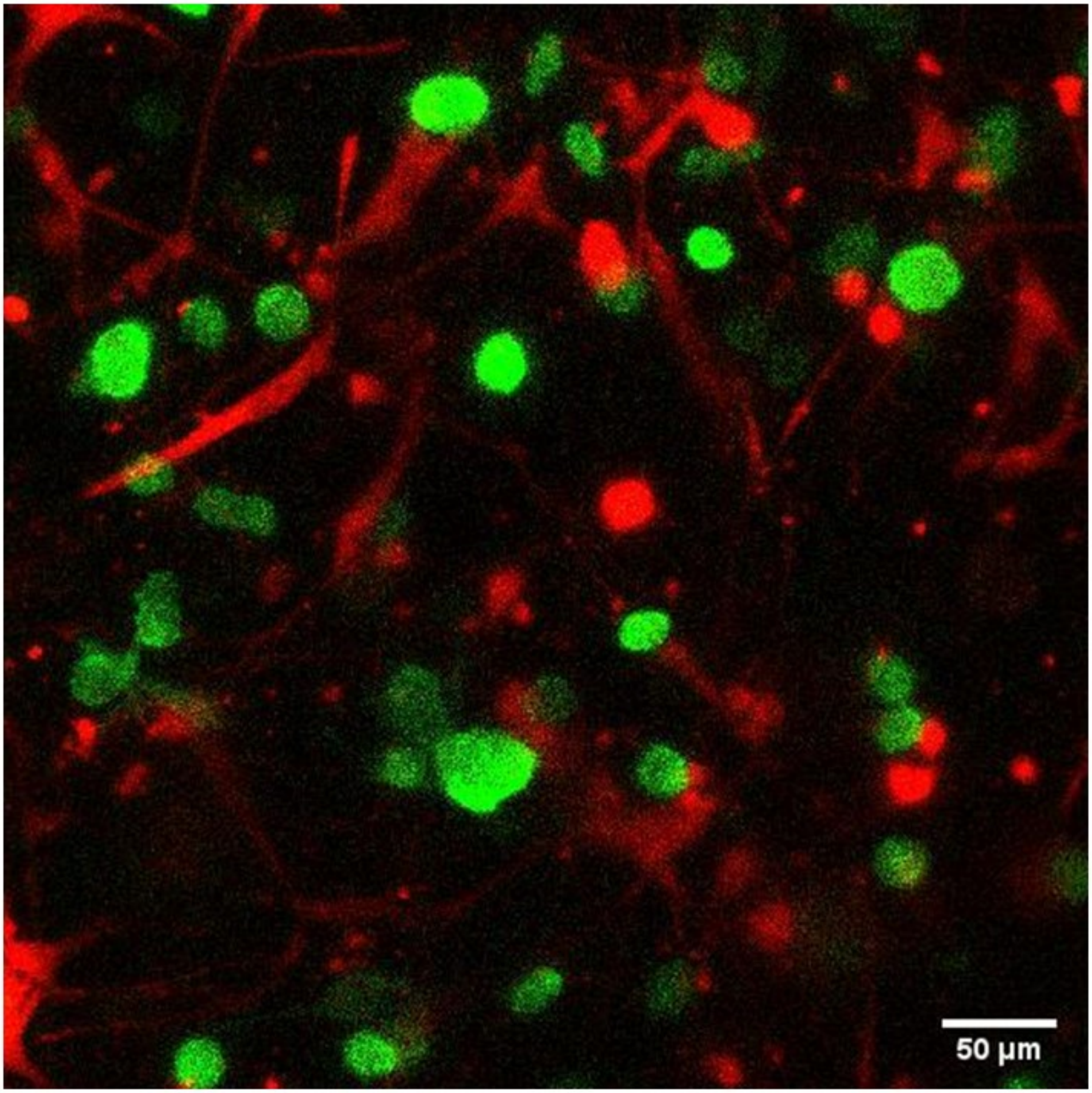

SI Video 1. MDA-MB-231 cells (green) are highly motile even when not in direct contact with fibroblasts (red). Scale bar is 50μm.

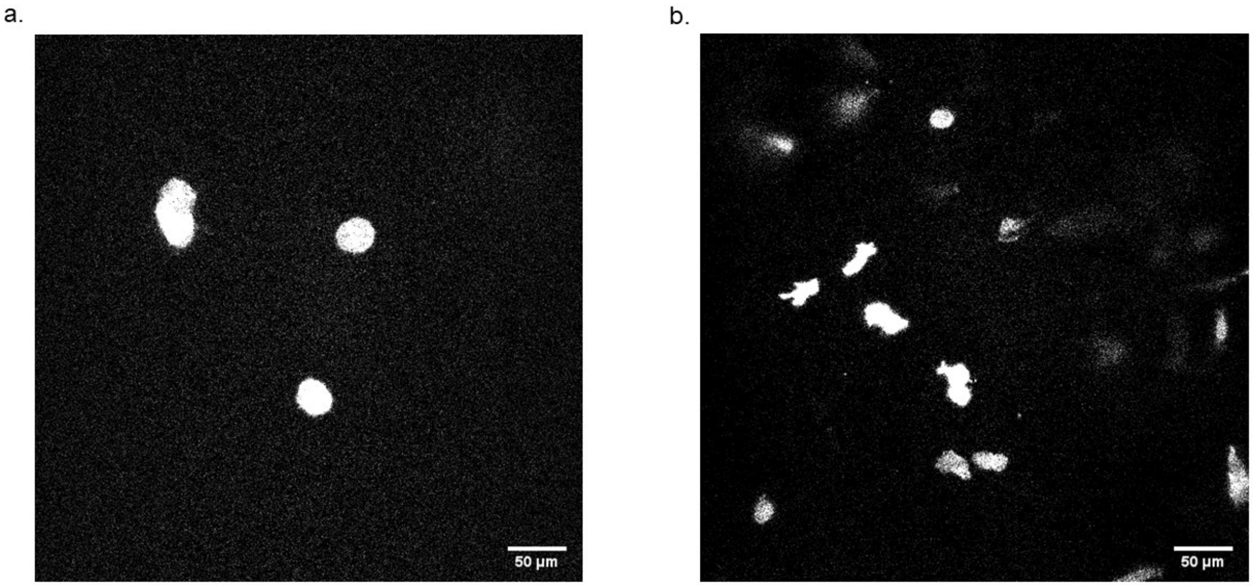

SI Video 2. a. Time-lapse video of MDA-MB-231 monoculture records cell migration from ∼24hrs-36hrs post cell embedment; b. Time-lapse video of MDA-MB-231 co-cultured with NHLF, recording cell migration from ∼24hrs-36hrs post cell embedment. MDA-MB-231s are Lifeact labelled. Channel of NHLF is not shown in the video. Scale bar is 50μm.

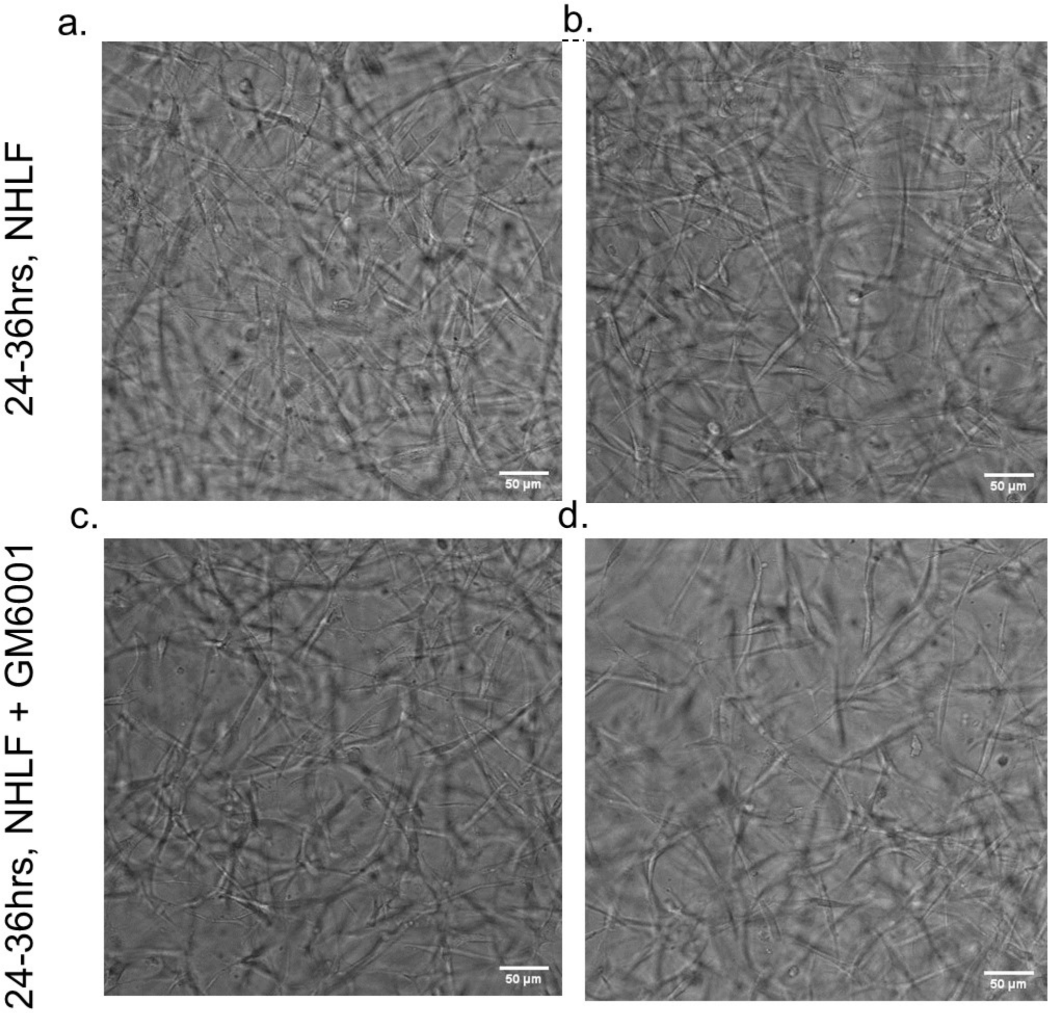

SI Video 3. Gel collapse induced by NHLF activities (Cells seeded with a density of 24000K/ml). a and b show gel compaction between ∼24hrs-36hrs post gel embedment. c and d show no gel compaction with 20μM GM6001 treatment at the same time frame. Scale bar is 50μm.

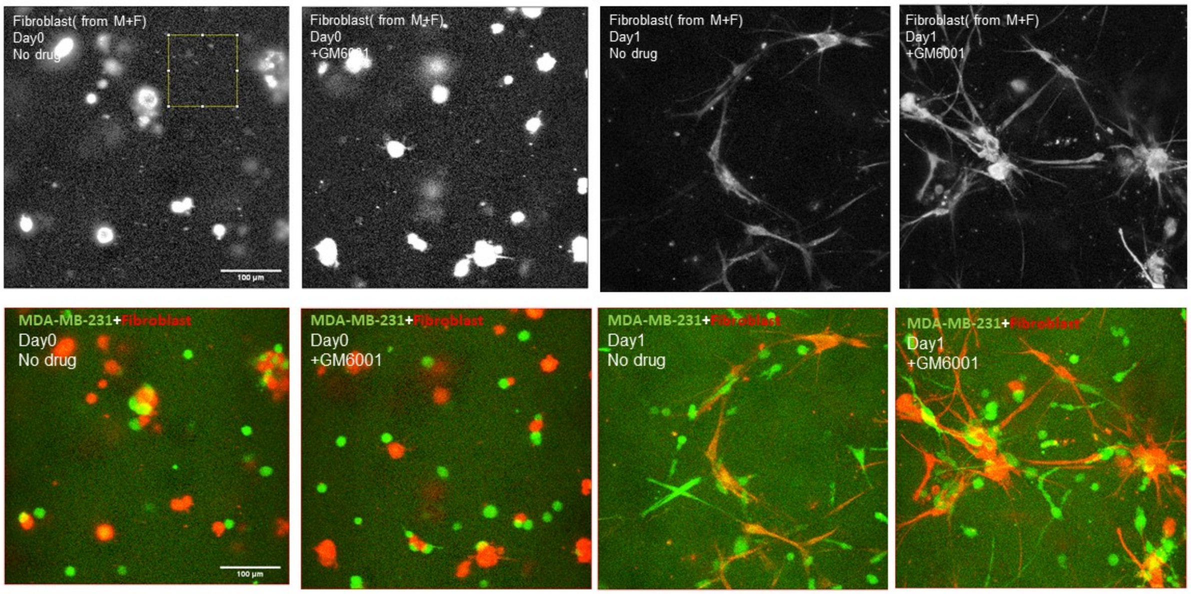

SI Video 4. Representative videos of gel contraction from MDA-MB-231(wildtype,GFP channel)+NHLF(red channel) coculture. Gel contraction is strong in the beginning of day0 right after gel embedment when fibroblasts extend cell body, regardless of GM6001 treatment. MDA-MB-231 from M+F condition(no drug)on day1 demonstrate fast and active migration compared with gel contraction trend. Scale bar is 100μm.
